# Continuous mass photometry by single-molecule trapping

**DOI:** 10.1038/s41592-026-03252-1

**Published:** 2026-10-26

**Authors:** Dan Loewenthal, Roi Asor, Stephen Thorpe, Jack S. Peters, Konstantin C. Zouboulis, Merve Kaplan, Jan Christoph Thiele, Jack Bardzil, Seham Helmi, Sean A. Burnap, Tiong Kit Tan, Weston B. Struwe, Justin L. P. Benesch, Philipp Kukura

**Affiliations:** 1 Department of Chemistry, Physical and Theoretical Chemistry Laboratory, University of Oxford, Oxford, UK; 2 Biochemistry Building, Kavli Institute for Nanoscience Discovery, Oxford, UK; 3 Department of Biochemistry, University of Oxford, Oxford, UK; 4 MRC Translational Immune Discovery Unit, Weatherall Institute of Molecular Medicine, University of Oxford, Oxford, UK; 5 CAMS Oxford Institute, Chinese Academy of Medical Sciences & Peking Union Medical College, University of Oxford, Oxford, UK

**Keywords:** Single-molecule biophysics, Analytical biochemistry, Interference microscopy, Nanoscale biophysics, Biophysical chemistry

## Abstract

The dynamic choreography of biomolecular interactions underpins the processes of life, but its direct observation remains challenging. Here we introduce continuous mass photometry, enabling hour-long, mass-resolved observation of individual biomolecules tethered to supported lipid bilayers, their complexes and interactions, with up to sub-kilodalton mass precision and millisecond temporal resolution. Our approach represents a quantitative time-resolved single-molecule measurement modality for studying complex biomolecular mechanisms in action.

## Abstract (web summary)

Continuous mass photometry enables high-resolution observation of biomolecular masses and mass changes.

## Main

Technologies capable of studying single biomolecules have transformed our understanding of biomolecular processes^1–3^. Nevertheless, one of the core goals of single-molecule biophysics—continuous, quantitative and time-resolved observation of the dynamics of biomolecular complexes—has remained challenging. Achieving this would reveal information key to understanding the molecular basis of function and regulation and the development of therapeutics, yet difficult to access in ensemble measurements: the sequence of biomolecular interactions, dwell time distributions and transitions that define biomolecular mechanisms.

Single-molecule mass measurements based on refractive index contrast^4–7^ promise a direct and quantitative way to study biomolecular interactions in a time-resolved manner. This is because mass is a universal and specific observable, directly reporting on biomolecular composition, while refractive index contrast is well suited for extended, time-resolved studies. Despite this promise, current approaches are constrained in observation time, operation in mechanistically relevant conditions, or both.

Cavity-enhanced methods^5^ achieve high sensitivity and even conformational detail in free-solution, although measurements are currently limited by fast transient passage times. In parallel, nanofluidic approaches^6^ have made substantial advances in extending free-in-solution observation times. However, scaling these methods to the sample complexity necessary for many biological interactions remains an area of active development. Mass photometry quantifies the mass of single proteins and their complexes by scattering-based readout of refractive index changes when molecules bind to a glass–water interface^8,9^. Although standard landing assays provide rapid mass quantification of heterogeneous mixtures, they have yet to demonstrate the ability to monitor subsequent interactions.

Supported lipid bilayers (SLBs) partially address these limitations by enabling dynamic mass measurements of tethered biomolecules through lateral diffusion and continuous background subtraction^10,11^. This approach facilitates mass-based tracking and quantification of interactions between surface-tethered molecules, and between surface and solution-phase molecules^12^. However, SLB-based observations are typically restricted to a few seconds because molecules exit the field of view. While such observation times may suffice for determining fast dynamics, they are insufficient for monitoring multi-step processes or substantially enhancing mass precision through repeated measurements of the same molecule.

**Fig. 1:**
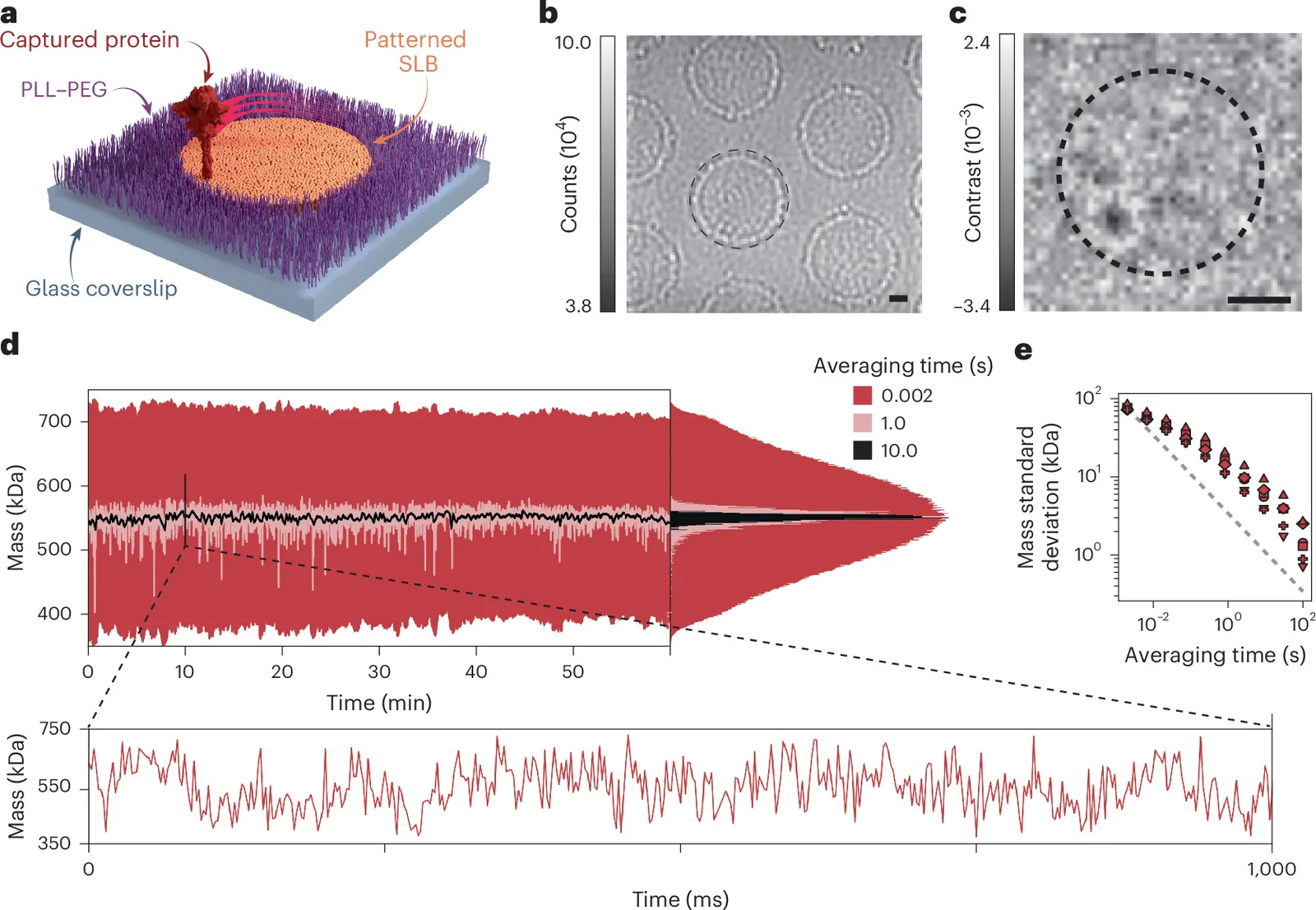
Hour-long mass monitoring of a single protein. **a**, Schematic of a cMP experiment. Molecules are not drawn to scale. **b**, Mass photometry image of patterned SLB traps. **c**, Median processed mass photometry image revealing a diffusing SARS-CoV-2 trimeric spike glycoprotein. Scale bars, 1 µm. **d**, Top: cMP mass versus time trace of a single diffusing SARS-CoV-2 spike glycoprotein. Bottom: 1-s zoom at 2 ms averaging time. **e**, Mass precision as a function of averaging time for six different spike proteins. The dashed gray line represents $\frac{1}{\sqrt{N}}$ scaling expected from averaging uncorrelated measurements.

We reasoned that combining single-molecule trapping via patterned SLBs with mass photometry could overcome this limitation. In this approach, individual molecules are retained within the microscope’s field of view and isolated from spatially overlapping with other tethered biomolecules, while allowing them to interact with species from solution. To achieve this, we prepare geometrically patterned SLBs surrounded by dense poly-L-lysine polyethylene glycol (PLL-PEG) brushes using photolithography (Fig. 1a,b and Supplementary Fig. 1). The SLBs contain 1% of nickel-nitrilotriacetic acid functionalized lipids that interact strongly with His-tagged proteins^12^, tethering them to the surface while allowing lateral diffusion. The combination of SLB and PEG provides excellent surface passivation except for the species of interest, allowing the presence of other biomolecules in solution without perturbing the mass measurement. Following the addition of the target protein, in this case 6xHis-tagged severe acute respiratory syndrome coronavirus 2 (SARS-CoV-2) spike glycoprotein (536 ± 5 kDa), at 5 nM concentration and washing after 30 s, we find anywhere between zero and three spike glycoproteins bound to SLB traps (Fig. 1c and Supplementary Fig. 1).

Using commercial mass photometry instruments (wavelengths 488 and 520 nm and a field of view of ~200 and ~100 μm^2^, respectively), we observed that the diffusing species became immobilized (Supplementary Fig. 2) over the course of tens of seconds upon illumination. We suspect that this immobilization stems from photoinduced damage to the SLB, exacerbating imperfections in the SLB to expose a glass surface to which bilayer-tethered flexible spike proteins can immobilize, although more experiments are required to test this hypothesis. Upon moving to 638-nm illumination using a custom-built instrument, however, we could frequently monitor individual proteins for up to an hour before immobilization (Supplementary Figs. 3–5), although with the trade-off of a weaker optical contrast signal (0.9 × 10^−5^ kDa^−1^ compared with 3.0 × 10^−5^ at 520 nm and 3.9 × 10^−5^ at 488 nm). For an imaging frame rate of 497 Hz, an hour-long trajectory thus consists of ~1.8 million position and mass measurements, from which we can extract both mobility and molecular mass (Fig. 1d, Supplementary Figs. 3–5 and Supplementary Movie 1). Moreover, as a direct consequence of extended observation, we can improve the mass precision by signal averaging at the expense of temporal resolution^13,14^, reaching ~1 kDa for a 536-kDa protein with 100-s temporal averaging (Fig. 1e).

**Fig. 2:**
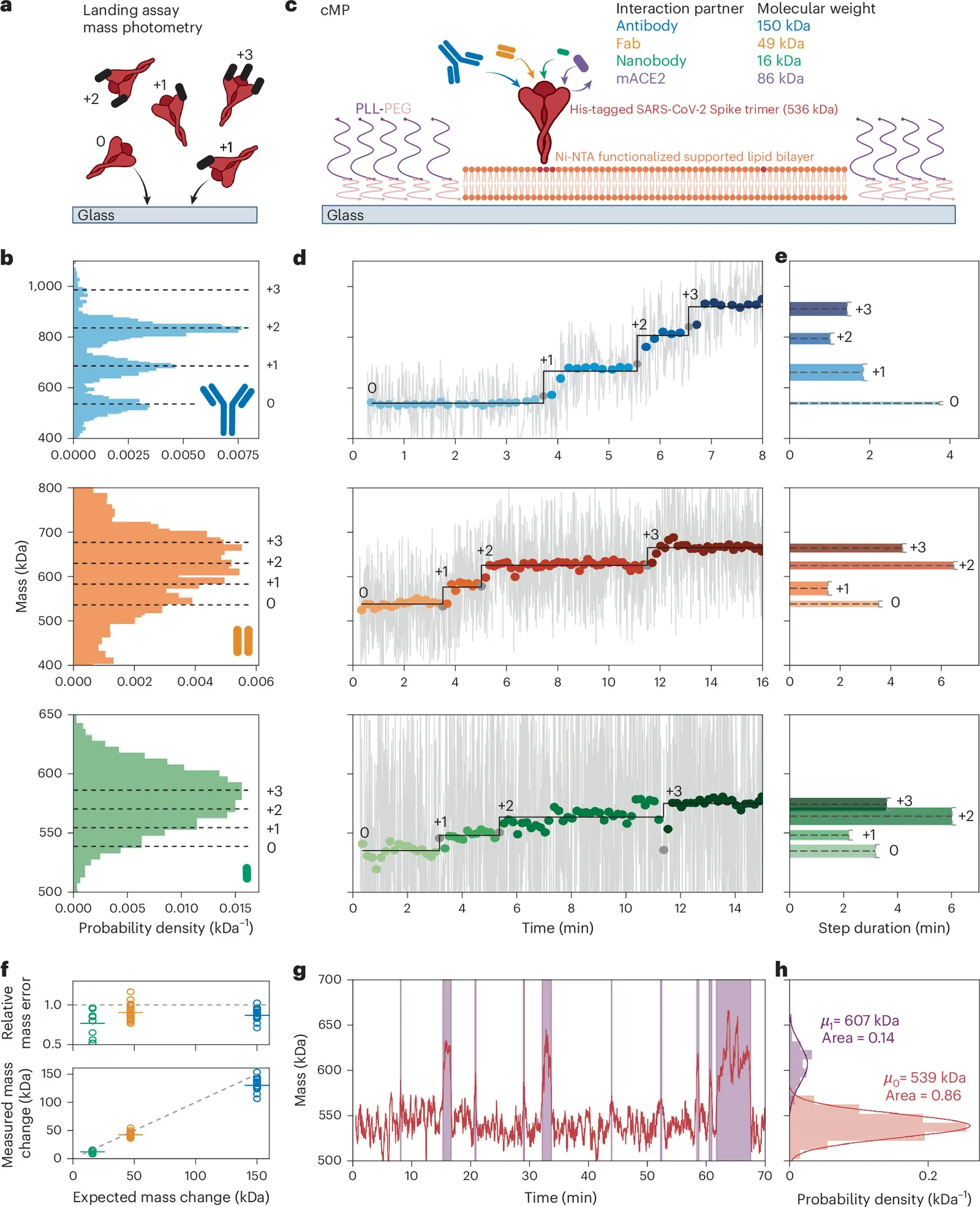
Real-time monitoring of single-molecule interactions by step changes in mass. **a**, Schematic of a standard mass photometry (MP) landing assay. **b**, MP histograms for SARS-CoV-2 spike mixtures (top: antibody, middle: Fab, bottom: nanobody). **c**, Schematic of the cMP experiment. **d**, Mass versus time traces (gray: raw, scatter: 10 s averaged) of a single confined SARS-CoV-2 spike protein binding to: Top: antibody. Middle: Fab. Bottom: nanobody from solution. **e**, Corresponding quantification of the observed molecular masses and their dwell times. **f**, Measured versus expected mass changes and relative mass error (antibody: 17, Fab: 25, nanobody: 11). **g**, Mass versus time trace of a SARS-CoV-2 spike protein interacting transiently with mACE2. **h**, Gaussian fits to the states found in **g**.

To assess the ability of continuous mass photometry (cMP) to detect mass changes caused by biomolecular binding, we measured the interactions between SARS-CoV-2 spike trimers and a series of ligands with progressively smaller masses: IgG antibodies (150 ± 1 kDa), IgG fragment antigen-binding regions (Fabs) (49 ± 1 kDa) and nanobodies (16,480 ± 1 Da). In standard mass photometry (Fig. 2a), distinct molecular species can be resolved for the antibody interaction. However, for the Fab and nanobody (Fig. 2b), these species become unresolvable, as the measurement error for the masses of the coexisting species is comparable in magnitude to the mass spacing between them.

For trapped molecules (Fig. 2c), a single molecule that binds and unbinds its interacting partner from solution yields different mass measurements over time. As a result, the observed masses appear sequentially rather than simultaneously (Fig. 2d). Thus, the challenge now becomes one of detecting changes in molecular mass, as opposed to resolving a mixture. This principle is analogous to super-resolution fluorescence microscopy, where fluorophores are switched on and off at different times so they can be localized independently. Similarly, here, the tethered molecule interacts with other molecules in solution, giving rise to temporally separated, distinct molecular states whose masses can be resolved independently. Once states are identified, their dwell time and average mass can be quantified (Fig. 2e). In each case, we observe a maximum of three binding events, corresponding to the three binding sites of the spike trimer (Fig. 2d,e and Supplementary Figs. 6–8). The associated mass changes scale well with the expected mass of the ligand, subject to a ~15% offset consistent with an expected reduction in interferometric contrast stemming from the distance of the receptor binding domain of the spike trimer from the SLB^12^ (Fig. 2f, Supplementary Fig. 9 and Supplementary Note 1).

The chosen interaction partners exhibit tight binding associated with a slow off-rate, directly visible from the mass versus time traces showing no evidence for ligand unbinding on the >10-min timescale^15,16^. To demonstrate dynamic (un)binding, we chose the interaction with a monomeric variant of the spike receptor, ACE2 (angiotensin-converting enzyme 2, mACE2, 85 ± 1 kDa). mACE2 was chosen to challenge the technique, featuring lower mass and faster unbinding kinetics compared with dimeric ACE2. Individual traces now show binding followed by unbinding events (Fig. 2g,h and Supplementary Fig. 10), in good agreement with the expected mass change of mACE2 with a stochastic range of dwell times.

The average bound lifetime of 64 s is in good agreement with previously reported lifetimes ranging from 30 to 230 s using biolayer interferometry and magnetic tweezers^17^ (Supplementary Fig. 10). These results demonstrate that interactions are observable by tracking step changes in mass. Currently, our method can reliably track dynamics where the analyte starting mass is >200 kDa, with mass changes of >20 kDa and lifetimes on the order of seconds to tens of minutes. We anticipate rapid developments in all key aspects of this method, further expanding the scope of the technique. While in this work we measure one patterned SLB at a time, throughput could be increased in the future by using larger imaging areas, optimizing confinement geometries, and developing controlled SLB (un)loading strategies to use the mass photometry field of view more effectively. Optimization of SLB chemistry and laser pulsing will lower background and measurement noise, cutting the required averaging time for kilodalton precision and enabling even longer observation times. Finally, the use of a capture complex on the order of 200 kDa bridging the lipid and target will substantially improve the detection limit, now bound only by mass precision rather than background noise.

## Methods

### Stocks and reagents

Isopropanol (24137-M), silicone gaskets (GBL 103280), HEPES (H3375), magnesium chloride hexahydrate (M2670) and KCl (P9541) were purchased from Merck Life Science UK Limited. SARS-CoV-2 nanobodies were bought from Nanotag Biotechnologies (cat. no. N3505). Glass slides (11836933) were purchased from Fisher Scientific. Lipids (1-palmitoyl-2-oleoyl-*sn*-glycero-3-phosphocholine, POPC: 850457, 1,2-dioleoyl-*sn*-glycero-3-phosphoethanolamine-*N*-[methoxy(polyethylene glycol)-550], DOPE-PEG550: 880530C, 1,2-dioleoyl-*sn*-glycero-3-[(*N*-(5-amino-1-carboxypentyl)iminodiacetic acid)succinyl] (nickel salt), DGS-NTA(Ni): 790404P) were purchased from Avanti Polar Lipids. PLL(20)-g[3.5]-PEG(2) was purchased from SuSoS surface technology.

### Liposome preparation

Similarly to our previously described protocol^10^, liposomes were prepared by dissolving POPC, DOPE-PEG550 and DGS-NTA(Ni) in chloroform and mixing them in a clean glass tube at a 97–2–1 molar ratio (corresponding to 500 μM total lipid concentration in 0.5 ml of buffer). The mixture was then dried under a constant nitrogen stream via rotary evaporation and further dried under vacuum for 1 h at room temperature (22–23 °C). A total of 500 μl HKS-150 (20 mM HEPES, pH 7.4, 150 mM KCl) was added to the dried lipids and the mixture was covered with parafilm, incubated at 50 °C in a water bath for 1 h, briefly vortexed and stored overnight at room temperature. The resulting liposome mixture was transferred into a 1.5-ml Eppendorf tube and kept in an ice-water bath and sonicated using a 3-mm probe at 25% amplitude with a 1 s on–3 s off sonication cycle (Sonics & Materials) for 10 min (that is, 40 min in total). Sonicated liposomes were then spun at 21,130*g* for 30 min at 4 °C, and the supernatant was collected in an Eppendorf tube, stored at 4 °C and used within 30 days.

### Patterned SLBs

Patterned SLBs were made based on a published protocol with some adjustments^18^. In brief, glass coverslips were placed in a metal rack and cleaned by sonication for 5 min in Milli-Q water followed by sonication for 5 min in a solution of 50/50 Milli-Q water/isopropanol. The coverslips were then dried with nitrogen and then plasma cleaned using a Zepto plasma cleaner (Diener electronic) operating at 40% power for 5 min. The coverslips were then rinsed with Milli-Q water and dried with nitrogen. The coverslips were then fitted with gaskets and were incubated with a 2 μg ml^−1^ PLL(20)-g[3.5]-PEG(2) solution in Milli-Q water for 30 min. Before being subjected to photolithography, coverslips were thoroughly rinsed with Milli-Q water and dried with nitrogen. The coverslips were then exposed to deep ultraviolet light in a mask aligner (Suss MJB4, HgXE 500 W source) for 60 min using a home-made photolithography mask. The coverslips were then thoroughly rinsed with Milli-Q and dried with nitrogen. These were kept at −20 °C for up to 3 months before use. Before every experiment, the coverslips were brought to room temperature then exposed to 25 μl fusion buffer (20 mM HEPES, pH 7.4, 150 mM KCl and 2 mM MgCl_2_) and 5 μl of liposome solution in a silicone gasket. After bilayer formation (monitored by mass photometry), which took around 5–10 min, the gaskets were washed with phosphate-buffered saline (PBS) five times by replacing the full volume of solvent in the gasket. The surfaces were blocked with 0.1 mg ml^−1^ PLL–PEG for 30 min unless otherwise noted and then washed again ten times with PBS by solvent volume replacement.

### cMP optical setup

Measurements were performed on a home-built mass photometry setup^4^ equipped with a red diode laser (Lasertack, 638 nm, 1 W) and a Blackfly 17S7M-C, FLIR camera, where acousto-optic deflector scanning was controlled using a Digilent Analog Discovery 2 device (Digilent). The setup was operated using a home-built LabVIEW script.

### Data acquisition

#### cMP

Mass photometry images were measured in a field of view (FOV) of approximately 6 × 6 μm, which was centered on a ~3.5-μm-diameter trap. The area was continuously scanned, and images were taken using an exposure time of 1,700 μs at 497 Hz for the long tracking experiments. For the step change and transient binding experiments, the acousto-optic deflector scanning was pulsed to position the beam further from the trap when the camera is not exposed, and the frame rates were 250 Hz and 10 Hz, respectively, with the same exposure time per frame.

#### cMP—long tracking experiments

SLBs were not blocked before protein addition. Spike was added at a 5 nM concentration in solution for capture and washed within 30 s for a capturing occupancy of zero to two particles per confined SLB. The trap was illuminated in the cMP setup with continuous illumination at a 497-Hz frame rate and 1,700-μs exposure time.

#### cMP—step change experiments

Spike trimers were added at a 5 nM concentration in solution and washed within 30 s for a capturing occupancy of zero to two particles per confined SLB. The trap was illuminated in the cMP setup with pulsed illumination at a 250-Hz frame rate and 1,700-μs exposure time. For the first 3 min in each experiment, no interaction partner was added in order to establish a baseline mass. At the 3-min mark, an antibody, Fab or nanobody was added. Antibodies were added as 100 nM by a 0.75:99 dilution from a 13.3 μM stock, and every 5 min the solution was mixed with a pipette. Fabs were added to a final concentration of 100 nM by a 10:90 dilution from a 1 μM stock. Subsequently, every 5 min, an additional 10 μl of the fab stock was added, increasing the concentration to facilitate binding within the measurement period. Nanobodies were added to a final concentration of 100 nM by a 10:90 dilution from a 1 μM stock. Subsequently, every 5 min, an additional 10 μl of the nanobody stock was added, increasing the concentration to facilitate binding within the measurement period.

#### cMP—transient binding experiments

Spike trimers were added at a 5 nM concentration in solution, and washed within 30 s for a capturing occupancy of zero to two particles per confined SLB. The trap was illuminated in the cMP setup at pulsed illumination at a 10-Hz frame rate and 1,700-μs exposure time. For the first 3 min in each experiment, no interaction partner was added in order to establish a baseline mass. At the 3-min mark, 210 nM of mACE2 was added while spike was continuously measured.

#### Landing assay mass photometry

Landing assay mass photometry measurements were performed on a Refeyn TwoMP at the ‘Regular’ FOV (10.9 × 4.3 µm and 498 Hz). They were performed on microscopy slides that were cleaned by consecutive sonication in Milli-Q water, followed by 50/50 Milli-Q water/isopropanol. The slides were fitted with silicone gaskets before measurement, and the total volume of protein solutions was 20 μl. Interaction mixtures were incubated at equimolar concentrations at 25 nM for 10 min, then measured as a 1:1 dilution with PBS in the mass photometry gasket for 60 s.

### Data processing

#### cMP

cMP measurements were analyzed using an in-house Python script^10,12^ adjusted for single trap analysis (Supplementary Fig. 11). In brief, proteins were detected using a Laplacian of Gaussian threshold filter on a median processed mass photometry movie, revealing the much smaller protein signal (~0.5%; Fig. 1c) compared with the raw image^10,11^. Detected proteins were then fitted to an analytical PSF function^10^ calibrated to the home-built mass photometry setup to determine the protein contrast. The fitted events were then filtered for single protein analysis. Lastly, the starting mass was set as the mean landing assay mass (Supplementary Figs. 12 and 13). The results were then presented as a running median of the raw fitted event masses or averaged mass values of consecutive time windows. For step detection, we implemented a step detection algorithm^19^ applying a maximum threshold of 10 steps per trajectory and minimum step size thresholds of 100, 30 and 8 kDa for the antibody, Fab and nanobody, respectively. The transient binding experiments (mACE2) were time averaged to 0.2 Hz, then fitted to a two-state hidden Markov model.

#### Landing assay mass photometry

Landing assay mass photometry data were analyzed using DiscoverMP software (V2024 R1). The frames were further averaged to navg = 15 (33.2 Hz) using the advanced software settings. Results are represented as the mean value of three technical replicates.

### Reporting summary

Further information on research design is available in the Nature Portfolio Reporting Summary linked to this article.

## Online content

Any methods, additional references, Nature Portfolio reporting summaries, source data, extended data, supplementary information, acknowledgements, peer review information; details of author contributions and competing interests; and statements of data and code availability are available at https://doi.org/10.1038/s41592-026-03252-1.

## Supplementary information

Supplementary Information Supplementary Figs. 1–13 and Note 1.

Reporting Summary

Supplementary Movie 1 Ten-minute mass photometry movie of a single SARS-CoV-2 spike glycoprotein diffusing in a patterned SLB.

## Data Availability

The raw data required to reproduce Figs. 1 and 2 are available via the University of Oxford Research Archive at https://doi.org/10.5287/ora-0z1qzq6wz.

## Code availability

The Python software and Jupyter notebooks required to reproduce Figs. 1 and 2 are available via the University of Oxford Research Archive at https://doi.org/10.5287/ora-0z1qzq6wz.
