# Supplementary material for: Continuous mass photometry by single-molecule trapping
Source: Nat Methods. 2026 Oct 26;23(11):2151–4. doi: 10.1038/s41592-026-03252-1 (PMC13736520; doi:10.1038/s41592-026-03252-1)
Supplement: Supplementary file 1 [file 41592_2026_3252_MOESM1_ESM.pdf]

nature methods

Supplementary information

https://doi.org/10.1038/s41592-026-03252-1

Continuous mass photometry by single-molecule trapping

In the format provided by the authors and unedited

Supplementary Information

Table of contents

Supplementary figure 1. Schematics for the experimental preparation of confined diffusion mass photometry experiments. ... 3

Supplementary figure 2. Illumination dependent immobilisation of confined SLB captured SARS-CoV-2 spike glycoproteins. ... 4

Supplementary figure 3. SARS-CoV-2 long tracking replicates. ... 5

Supplementary figure 4. Spatial distribution of confined diffusing proteins. ... 6

Supplementary figure 5. Characterisation of the confined diffusion mean squared displacement (MSD) ... 7

Supplementary figure 6. Antibody binding. ... 8

Supplementary figure 7. Fab binding. ... 9

Supplementary figure 8. Nanobody binding. ... 10

Supplementary figure 9. Step change quantitation. ... 11

Supplementary figure 10. Transient mACE2 binding. ... 12

Supplementary figure 11. Data analysis workflow. ... 13

Supplementary figure 12. Landing assay contrast to mass quantitation. ... 14

Supplementary figure 13. Mass correction factors distribution. ... 15

Supplementary note 1. Mass offset discussion. ... 16

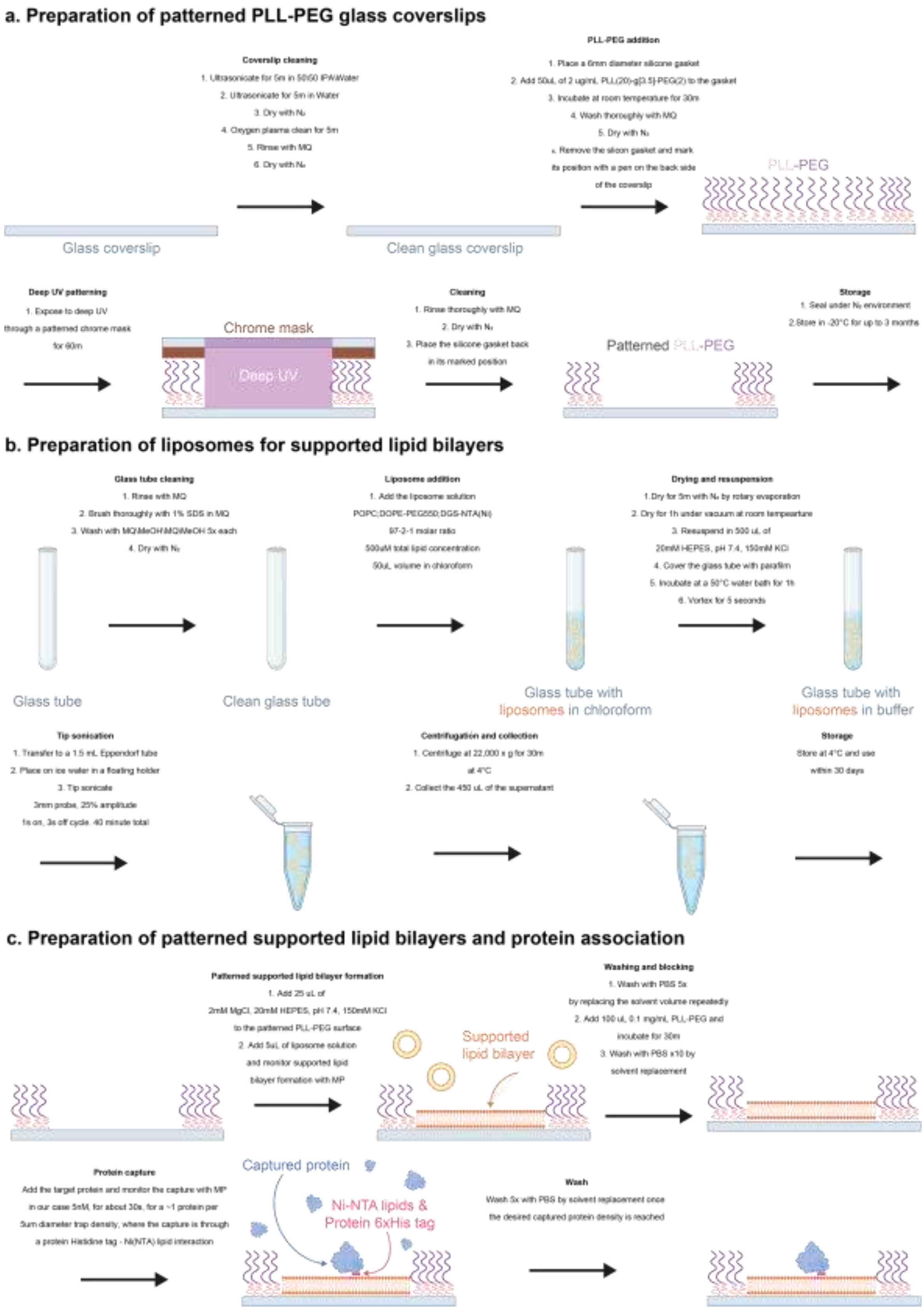

Supplementary figure 1. Schematics for the experimental preparation of cMP experiments.

(a) preparation of coverslips covered with patterned poly-L-lysine (PLL) – polyethylene glycol (PEG) through deep UV photolithography. **(b)** Preparation of liposomes solution for supported lipid bilayers. **(c)** preparation of patterned supported lipid bilayers and protein association.

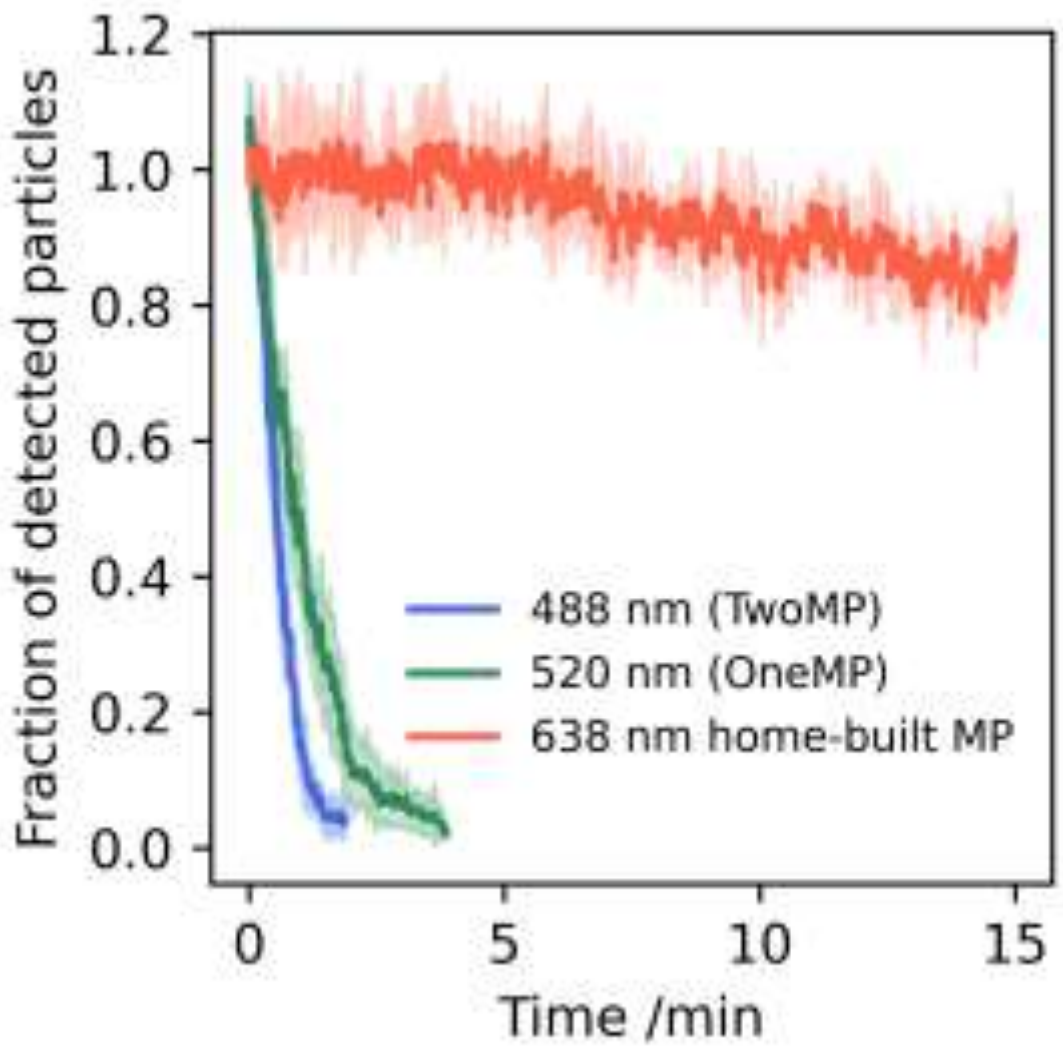

Supplementary figure 2. Illumination-dependent immobilisation of patterned SLB-captured SARS-CoV-2 spike glycoproteins. .

Spike glycoproteins were captured on 3.5 µm diameter patterned supported lipid bilayers (SLBs), and the number of detected (diffusing) proteins was monitored over time. Solid lines indicate the mean, and shaded areas represent the standard deviation across technical replicates (n = 4 for TwoMP, n = 4 for OneMP, and n = 3 for the home-built mass photometer). Each replicate included 4 patterned SLBs for the home-built and OneMP systems, and 6 patterned SLBs for the TwoMP system. For each replicate, traces were normalised to the average number of particles detected during the first 10 seconds of measurement. The field-of-view sizes were 16.9 × 12.0 µm² (TwoMP, Refeyn Ltd), 10.9 × 10.9 µm² (OneMP, Refeyn Ltd), and 10.0 × 10.0 µm² (home-built MP).

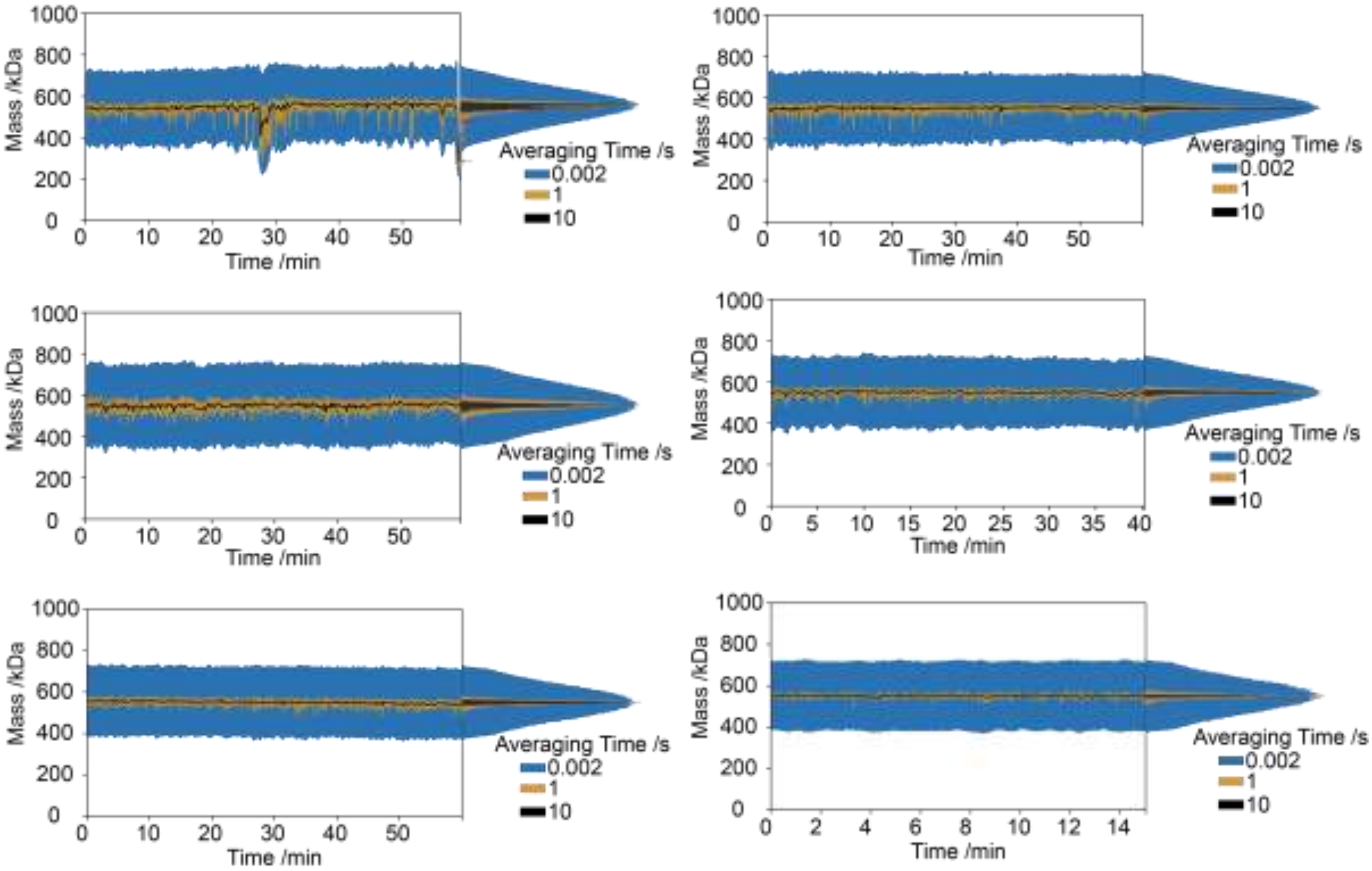

Supplementary figure 3. SARS-CoV-2 long tracking replicates.
Mass vs time traces of six replicates of a single SARS-CoV-2 glycoprotein diffusing across a patterned bilayer and measured with mass photometry. The patterns were of ~4 μm diameter supported lipid bilayer circles and the measurement was done with a 638 nm illumination at 497 Hz. Different averaging times show enhanced mass precision.

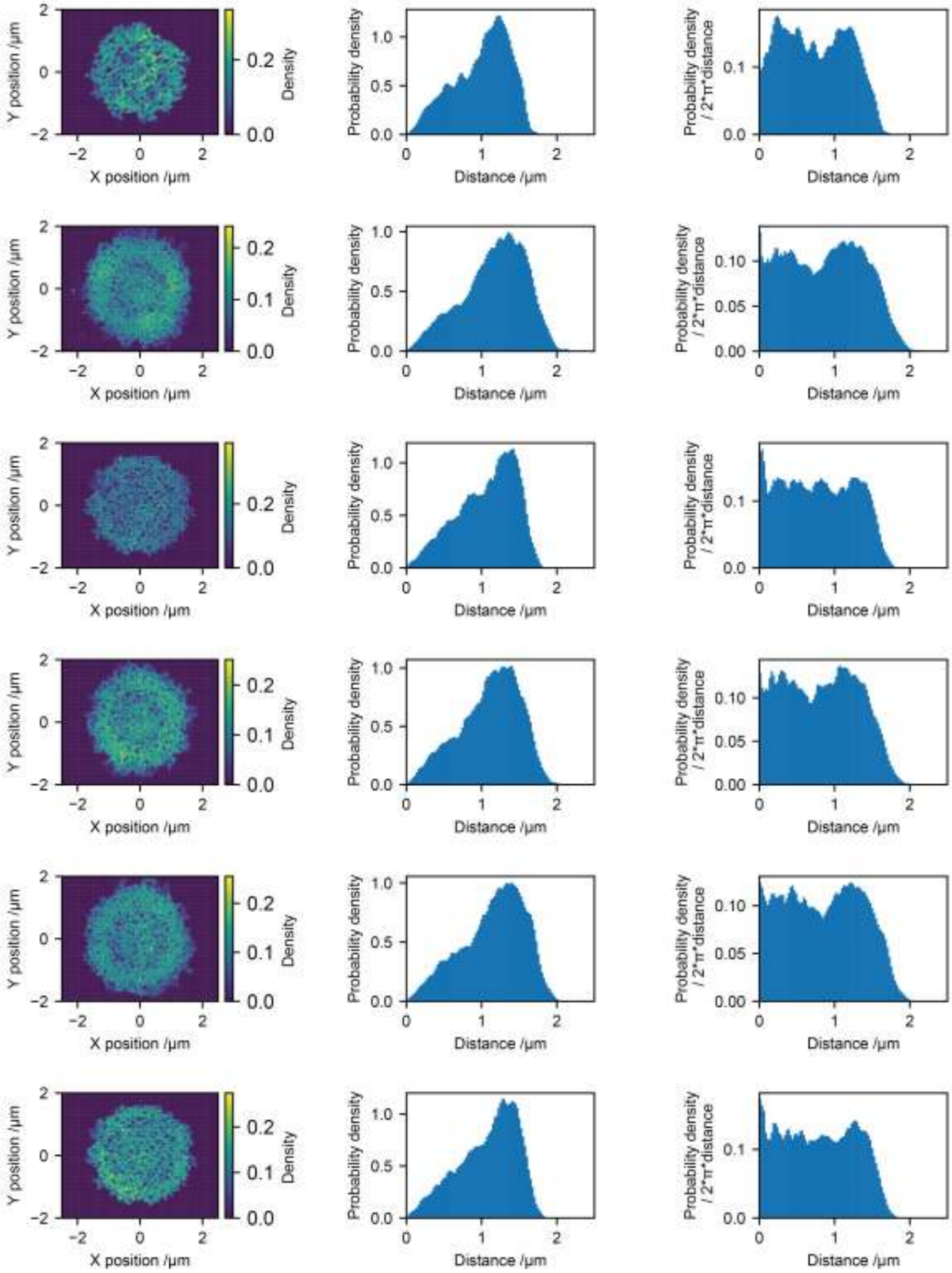

Supplementary figure 4. Spatial distribution of confined diffusing proteins.
Left to right: Heatmap of detected SARS-CoV-2 spike locations during long tracking showing confinement to the patterned supported lipid bilayer, probability density of the detected events as a function of distance from the centre of the patterned area, normalised probability density of detected events as a function of distance from the centre of the patterned area.

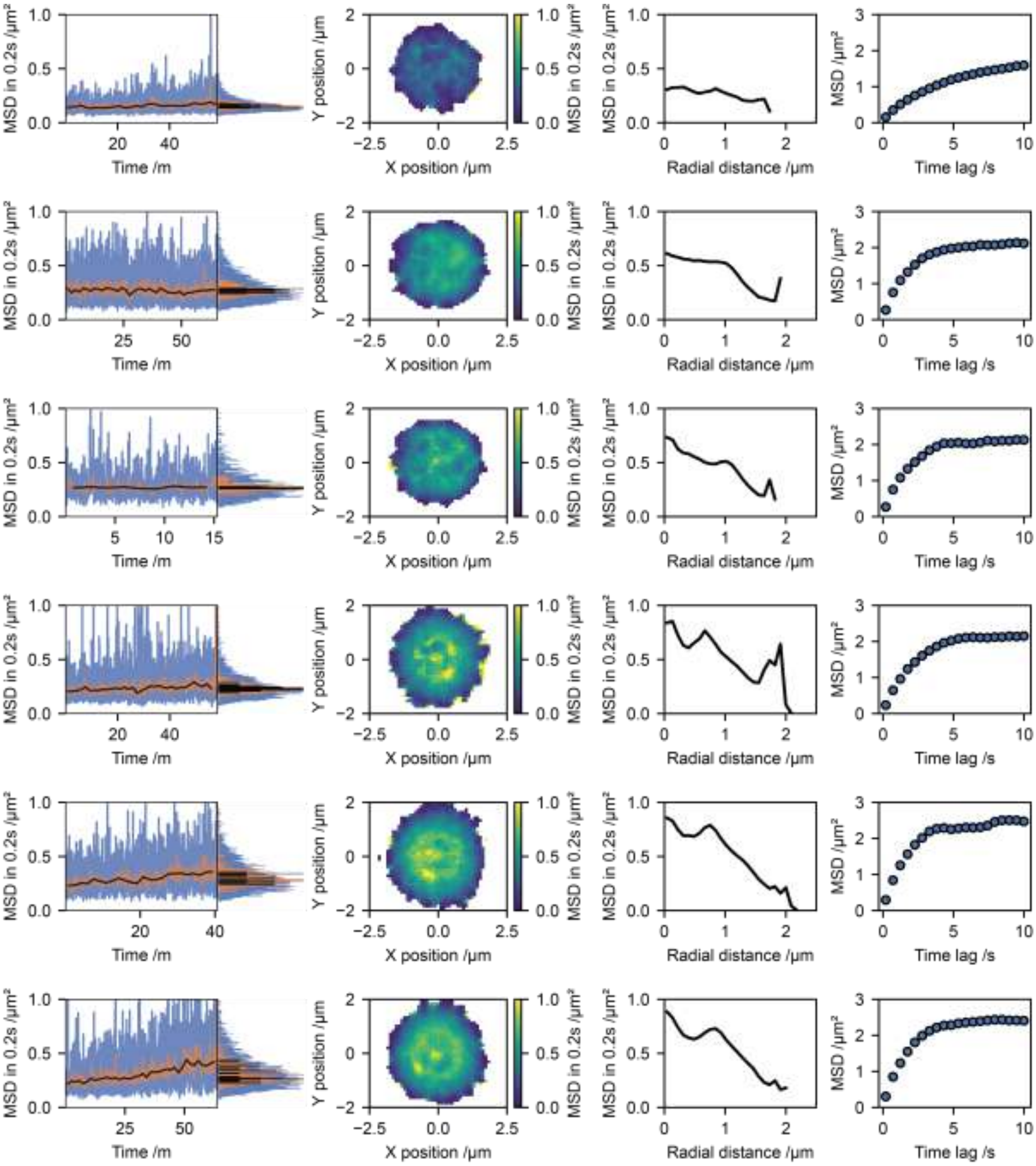

Supplementary figure 5. Characterisation of the confined diffusion mean squared displacement (MSD).

Left to right: Mean squared displacement (MSD) of a trapped particle in 0.2 seconds as either raw values (blue) or further time averaged for 1 second (orange) or 10 seconds (black); Heatmap of the distribution of MSD across the trap; Radial mean of observed MSD values; MSD values for different time lags.

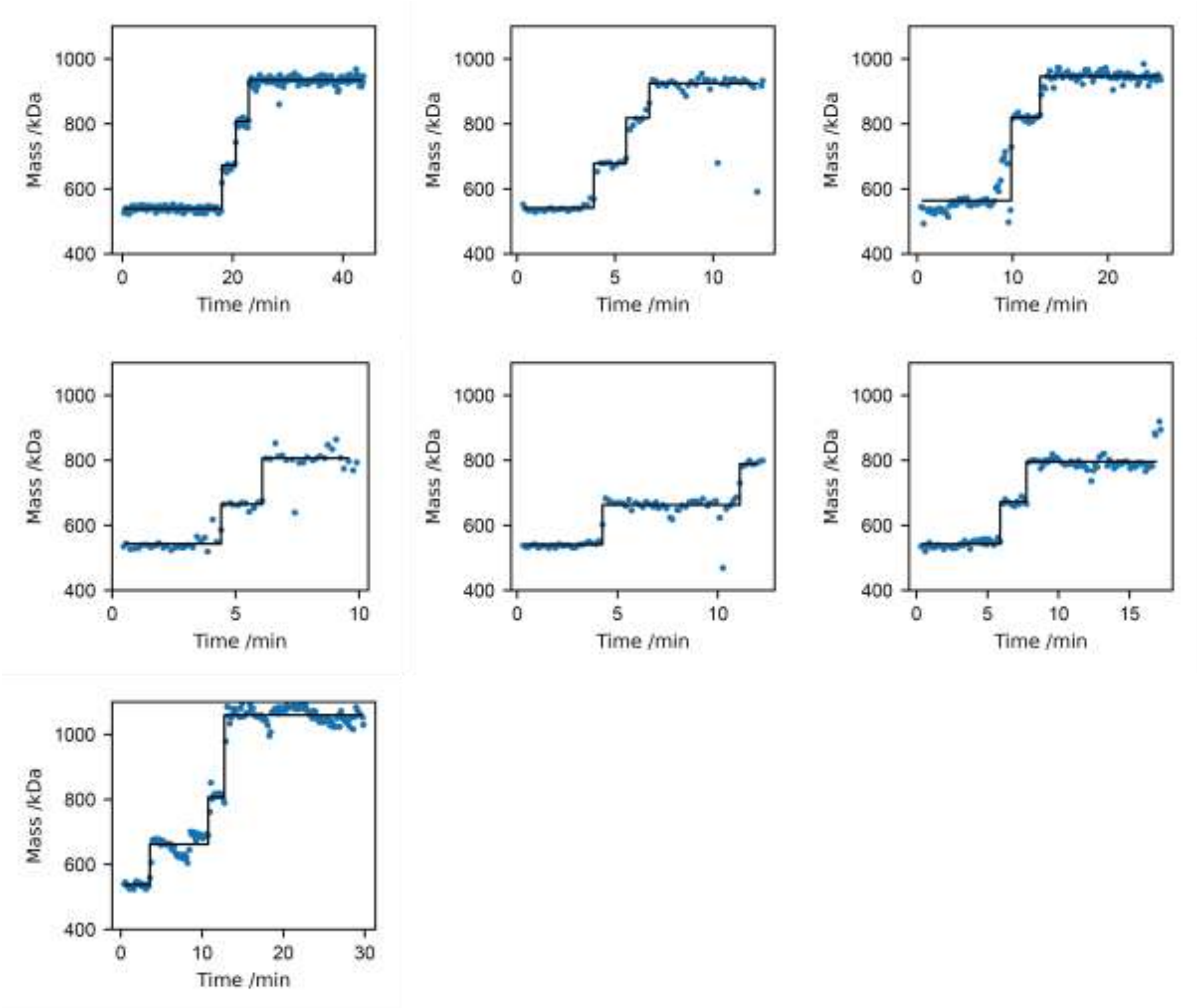

Supplementary figure 6. Antibody binding.
Mass vs time traces of seven replicates of stepwise antibody (150 kDa) binding to a SARS-CoV-2 spike trimer and their corresponding step function fit. Scatter points represent consecutive mass averaging windows of 10 seconds.

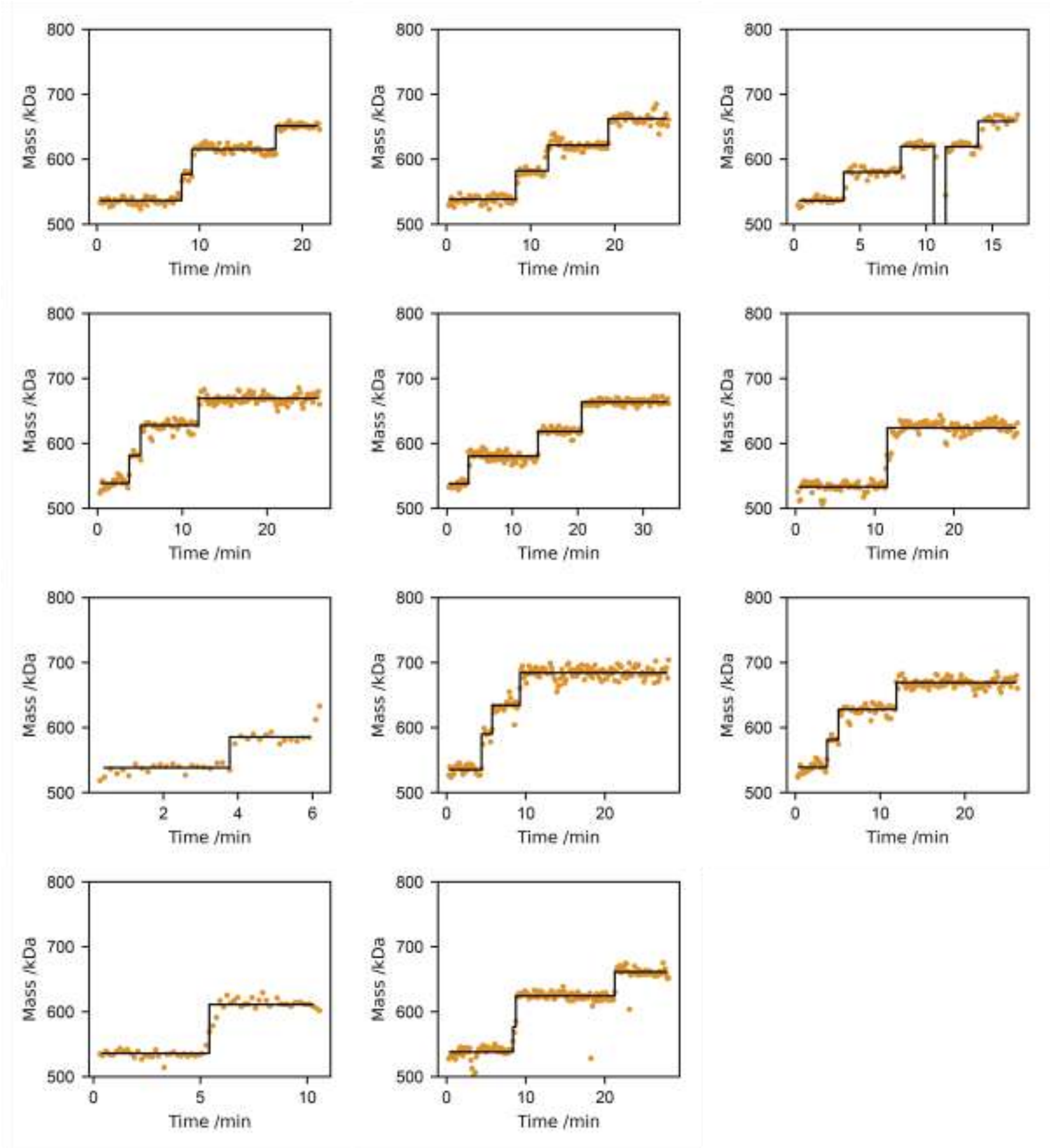

Supplementary figure 7. Fab binding.
Mass vs time traces of eleven replicates of stepwise Fab (49 kDa) binding to a SARS-CoV-2 spike trimer and their corresponding step function fit. Scatter points represent consecutive mass averaging windows of 10 seconds.

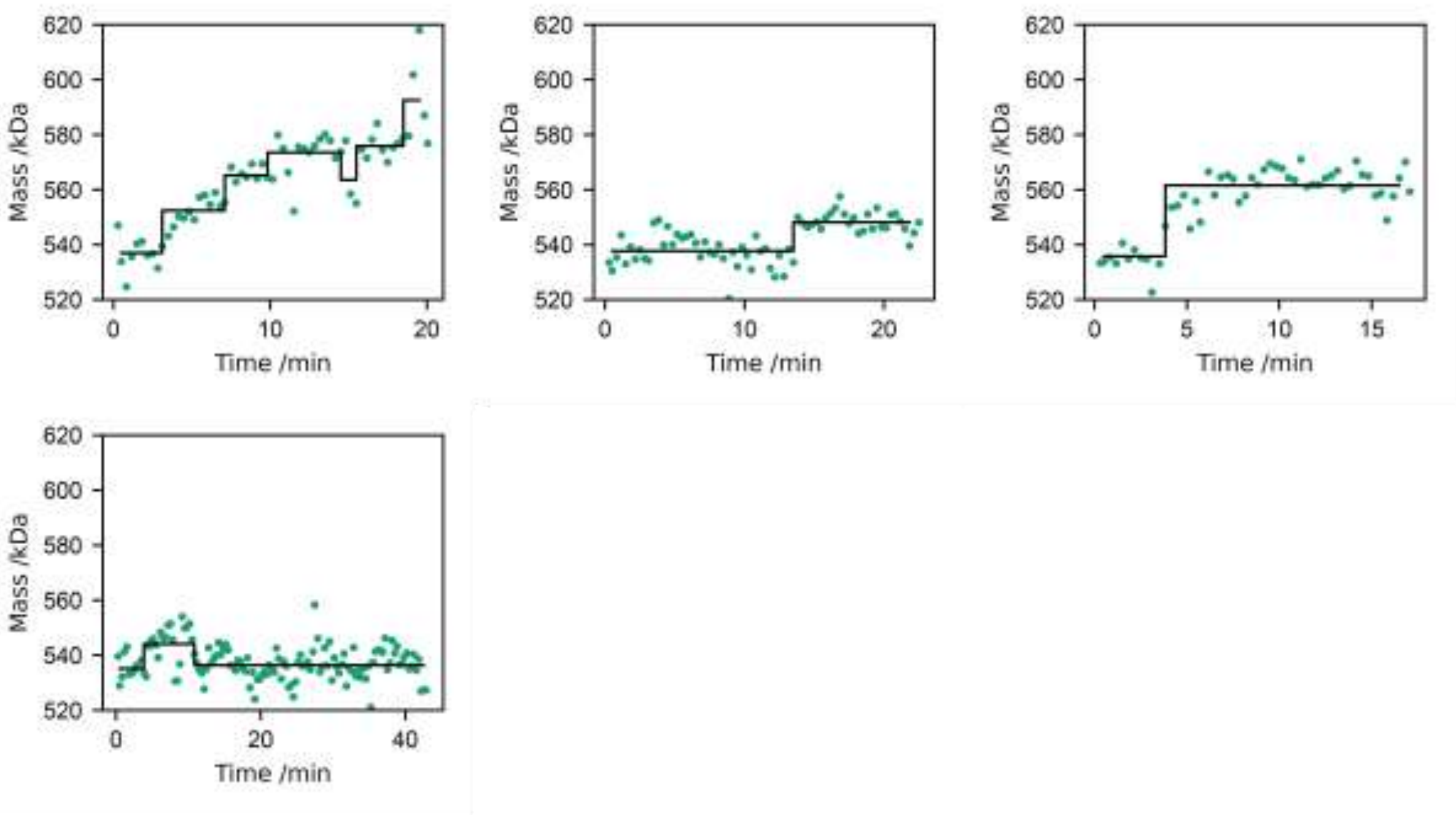

Supplementary figure 8. Nanobody binding.
Mass vs time traces of four replicates of stepwise nanobody (16 kDa) binding to a SARS-CoV-2 spike trimer and their corresponding step function fit. Scatter points represent consecutive mass averaging windows of 20 seconds.

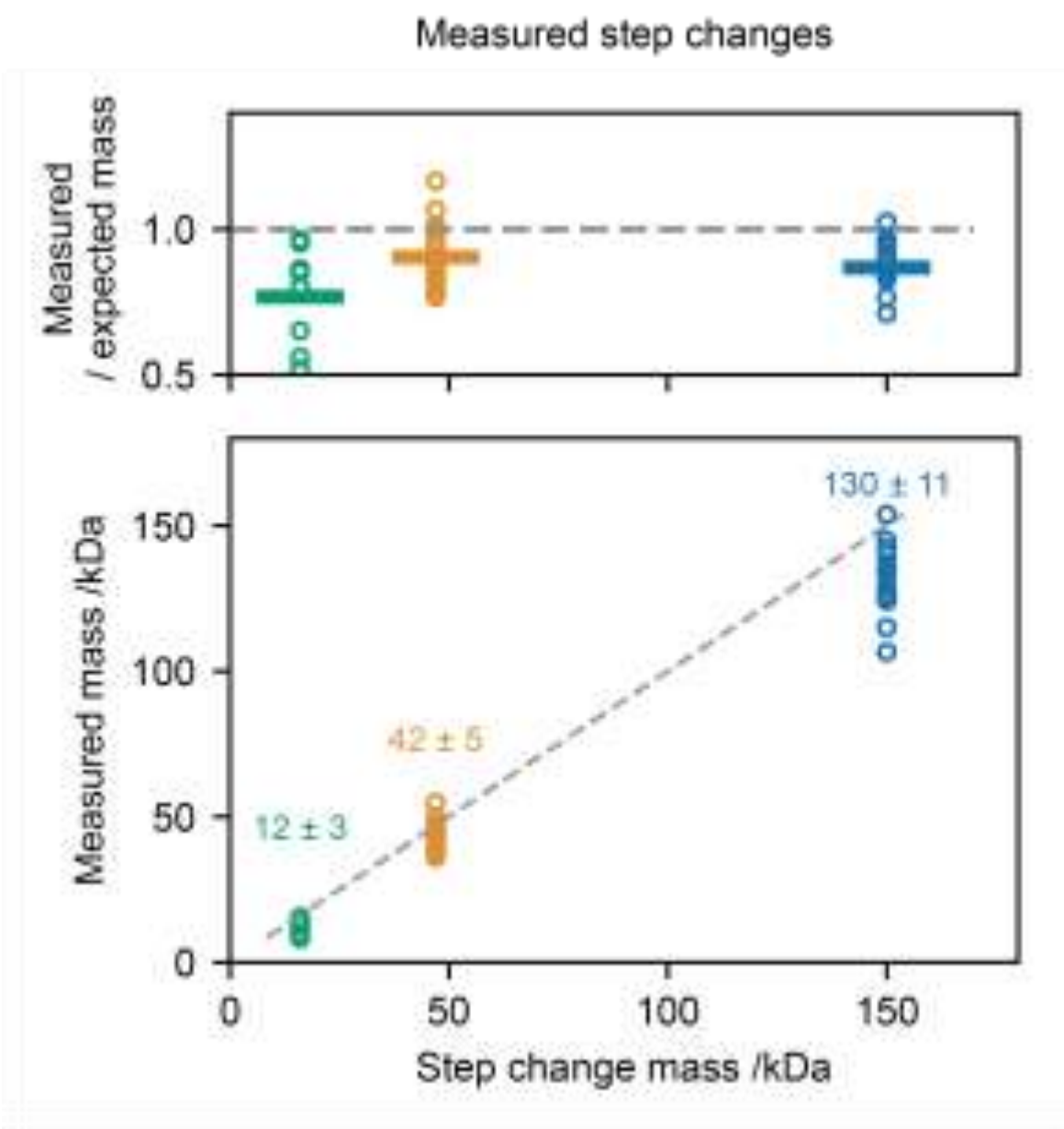

Supplementary figure 9. Step change quantitation.
Quantitation of the measured step changes for nanobody (green, n=8), Fab (orange, n=25) and antibody (blue, n=17) binding.

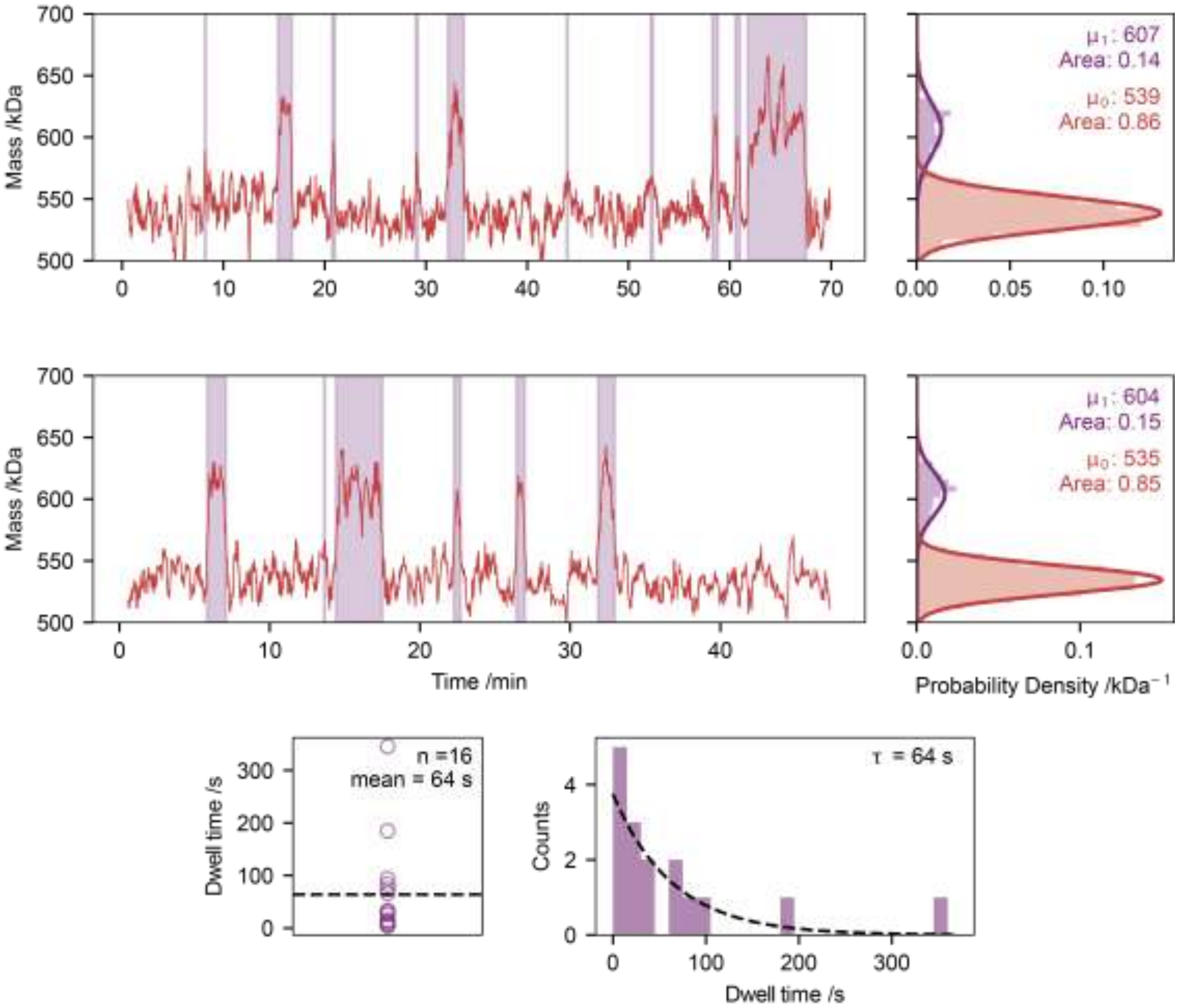

Supplementary figure 10. Transient mACE2 binding.
Mass vs time traces of two replicates of transient monomeric ACE2 binding to a SARS-CoV-2 spike trimer and a corresponding two state hidden Markov model fit. The red plot represents a mass averaged running median of 10 seconds and the purple areas represent the mACE2 bound state. The top plot is the same plot shown in figure 2 panels g-h. The bottom row shows the measured bound-state dwell time values, the mean dwell time, and a histogram of the dwell time distribution, together with the corresponding theoretical exponential decay curve based on the measured mean dwell time.

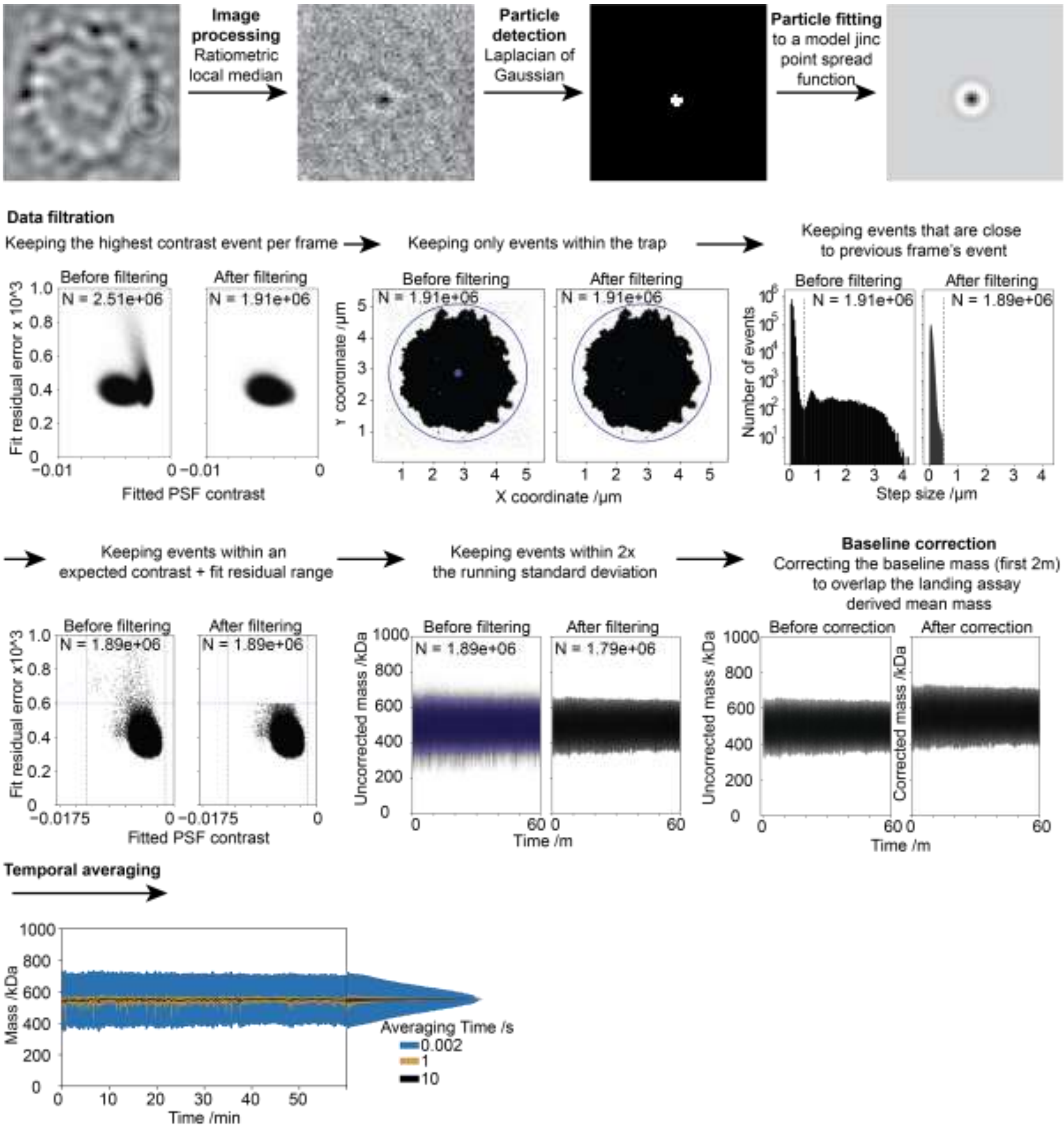

Supplementary figure 11. Data analysis workflow.
Schematic showing the data analysis workflow for cMP experiments starting from a raw mass photometry video up to a mass vs time trace.

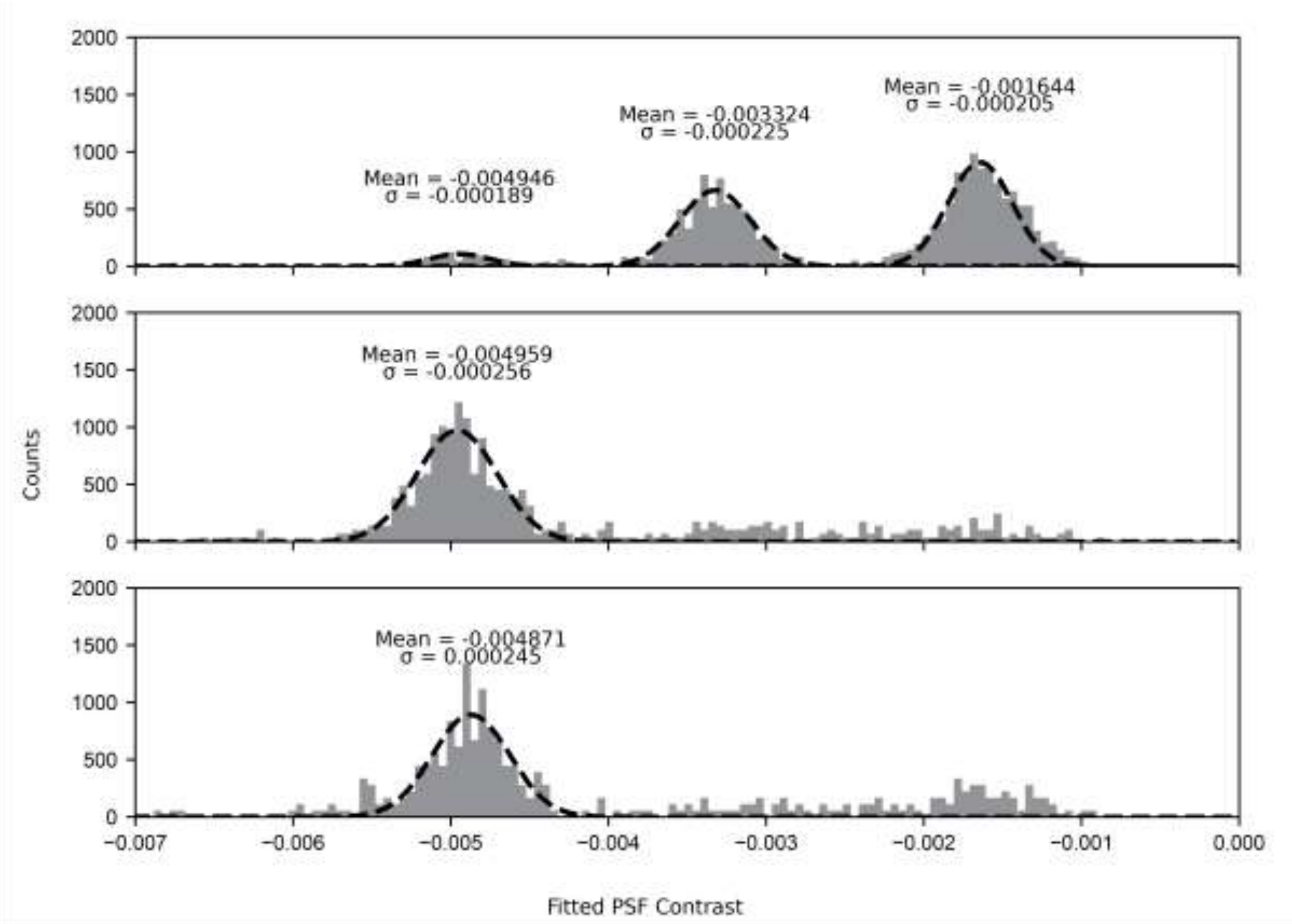

Supplementary figure 12. Landing assay contrast to mass quantitation.
Regular mass photometry landing assays measured at 250Hz, 2 ms exposure per frame at 638 nm and frame averaging time of 160ms for a mass calibrant (dynamin ΔPRD, top row) and SARS-CoV-2 spike (middle and bottom rows). Establishing a contrast to mass slope of -0.917E-6 [contrast/kDa] and an average SARS-CoV-2 spike mean mass of 536 kDa.

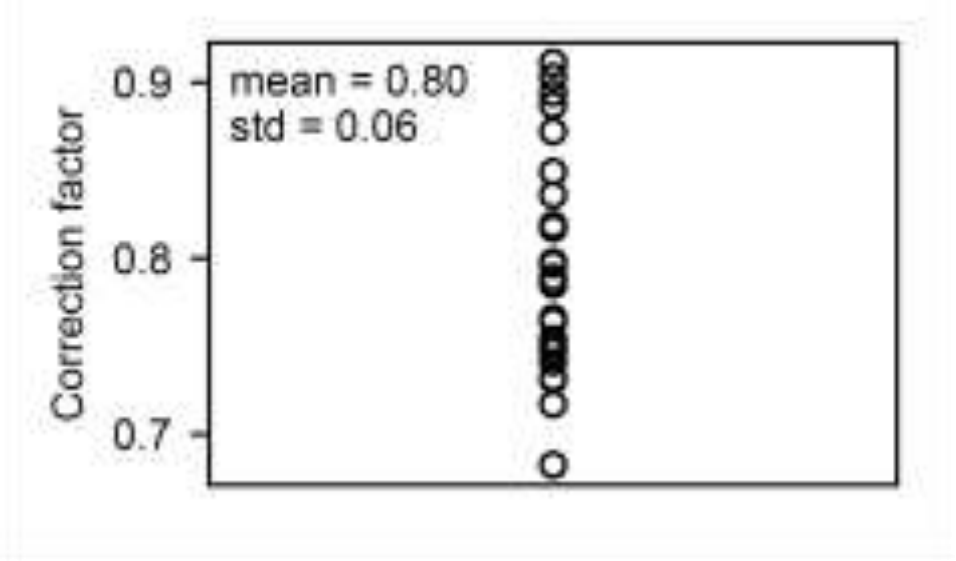

Supplementary figure 13. Mass correction factors distribution.
Scatter plot for the correction factors used across all of the spike traces in the manuscript in order to convert the estimated mass (fitted event contrast divided by landing assay derived mass slope) to the landing assay measured baseline mass. A correction factor of ~0.84 is to be expected, as shown previously (Asor, PNAS 2024). The replicate-to-replicate heterogeneity might stem from day-to-day mass slope differences, mass slope differences from measurement on different areas of the camera, diffusion differences in different traps and mass precision limitations given finite temporal averaging.

Supplementary note 1. Mass offset discussion

The origin of the small change (~15%) in the measured mass on the bilayer traps compared with measurements on glass, arises from variations of the interferometric contrast due to the dimensions of the spike glycoprotein, which extends up to approximately 25 nm above the glass-solution interface. At the same time, the contrast-to-mass conversion, and the microscope focus position during measurements were calibrated to maximise the signal at the glass water interface. This height variations alters the measured interferometric optical contrast owing to the added optical path difference between the reference light, reflected from the glass-solution interface and the scattered light from the spike (and the bound ligands). The overall measured contrast as a function of the z coordinates is given by,

$$C \propto 2\frac{|E_{scat}|}{|E_{ref}|}\cos\left(\phi_0 + \Delta OP(z)\right).$$

Where, $|E_{scat}|$ and $\left|E_{ref}\right|$ are the real amplitudes of the scattered and reflected fields and ϕ_0 is an intrinsic phase that includes the Gouy phase shift between the reference field and the field emitted by a point scatterer, as well as additional contributions from components of the imaging path such as the partial reflector at the back focal plane. Defocusing was optimised to provide the maximum contrast at the glass-solution interface, at these conditions $\phi_0(z = 0) \approx 0$, Where $z = 0$ is defined at the water glass interface. $\Delta OP(z)$, is the additional optical path accumulated by the scattered field owing to the scatterer position z nm above the interface. The optical path difference is given by $\Delta OP = \frac{4\pi \cdot \delta z \cdot n_m}{\lambda}$, and therefore the overall contrast is given by,

$$C \propto c_0 \cdot \cos\Delta OP(z) \ = c_0 \cdot \cos\frac{4\pi \cdot \delta z \cdot n_m}{\lambda},$$

where δ_{z_i}, n_m, λ are the *z* coordinate of the *i*-th trimeric subunit, the solution refractive index and the wavelength of the laser light source, respectively. Measurements of this height effect for the spike glycoprotein are shown in Figure S5 of Asor et.al https://doi.org/10.1073/pnas.2403260121
